# Comparative Assessment of Automated and Manual DNA Extraction Methods for the Genetic Analysis of Degraded Bone Samples

**DOI:** 10.3390/genes17070842

**Published:** 2026-07-22

**Authors:** Christina Amory, Walther Parson

**Affiliations:** 1 Institute of Legal Medicine, Medical University of Innsbruck, 6020 Innsbruck, Austria; christina.amory@i-med.ac.at; 2 Forensic Science Program, The Pennsylvania State University, University Park, PA 16802, USA

**Keywords:** bones, teeth, DNA extraction, automation, Maxwell FSC instrument, Dabney protocol, DNA quantification, massively parallel sequencing, mtDNA, Precision ID mtDNA Whole-Genome Panel

## Abstract

**Background:** Efficient DNA extraction from degraded skeletal remains is essential for forensic and ancient DNA analysis. The main aim of this study was to compare the performance of an automated DNA extraction system with a manual DNA extraction protocol when applied to challenging skeletal samples. Specifically, the automated Maxwell Forensic Sample Concentrator system was evaluated against a modified manual Dabney extraction protocol. **Methods:** DNA was extracted from skeletal material originating from twelve human individuals. Maxwell extractions using 50 mg and/or 100 mg of starting material were compared with Dabney extractions using 50 mg. DNA extracts were quantified using SD quants targeting nuclear DNA and two mitochondrial DNA fragments. Selected extracts were further analysed by mitochondrial DNA sequencing. **Results:** Both extraction approaches generated comparable DNA yields and sequencing results for moderately degraded samples. In the highly degraded samples analysed in this study, the Dabney protocol generally yielded higher nuclear and mitochondrial DNA quantities and was often associated with a higher sequencing performance. The Maxwell system nevertheless performed well for less degraded material and, in some cases, produced sequencing results comparable to Dabney. Maxwell extraction with 100 mg input was effective for better-preserved samples but was less consistent for highly degraded material. **Conclusions:** The efficiency of the extraction methods depended largely on the degree of DNA degradation. The findings of this study suggest that the Dabney protocol may be more suitable for heavily degraded skeletal remains, whereas the automated Maxwell system represents a practical and efficient option for less degraded samples. The choice of method therefore depends on the sample condition and the analytical objectives.

## 1. Introduction

The recovery of DNA from skeletal remains is a central component of forensic genetic and ancient DNA analysis. This is especially important when soft tissues are no longer available or when samples have been exposed to environmental conditions that accelerate DNA degradation [1,2,3,4,5,6,7,8]. Bones and teeth can preserve DNA over extended periods of time; however, the quantity and quality of recoverable DNA are highly variable and depend on factors such as age, burial environment, preservation state, microbial activity, and post mortem degradation. Consequently, the extraction method represents a critical step in the analytical workflow, especially for challenging samples that contain low amounts of fragmented endogenous DNA and potentially increased levels of inhibitors [6,7,8].

In forensic genetic routine work, automated extraction methods are generally preferred over manual protocols whenever applicable. Automation enables a more standardized workflow, reduces dependence on individual handling skills, saves hands-on time and overall costs, and may reduce the risk of individual handling-related contamination. These advantages are particularly relevant in high-throughput or casework-oriented laboratory environments, where reproducibility, traceability, and efficiency are essential. Commercially available automated systems also offer simplified handling and better integration into routine laboratory procedures compared with more labor-intensive manual extraction protocols.

Nevertheless, highly degraded skeletal samples remain analytically challenging. Manual silica-based extraction protocols, including the modified Dabney [1] method, have been specifically designed to maximize DNA recovery from highly fragmented DNA [6,7,8], such as that commonly encountered in ancient, historical, or otherwise compromised bone and tooth samples [1]. These methods are often more time-consuming and require considerable experience, but they may provide higher DNA yields and improved sequencing performance when sample preservation is poor. Therefore, the practical question is not only whether automation can replace manual extraction in general, but under which sample conditions automated methods provide results comparable to specialized manual protocols.

Among the various extraction methods available for degraded skeletal remains, an automated Maxwell workflow and the modified manual Dabney protocol were selected for comparison because they represent two fundamentally different strategies used in forensic DNA analysis. The Maxwell Forensic Sample Concentrator offers a standardized automated workflow suitable for routine laboratory use, whereas the modified Dabney protocol serves as a well-established manual reference method for highly degraded samples.

The present study evaluates the commercially available Maxwell Forensic Sample Concentrator system (Promega, Madison, WI, USA) in direct comparison with a modified manual Dabney extraction protocol [1]. Teeth and bone samples of different ages and preservation states were included, reflecting the variable preservation conditions commonly encountered in forensic and ancient DNA research [7,9]. The sample set comprised relatively well-preserved historical samples as well as highly degraded archaeological material. The automated workflow was evaluated using two different input amounts, 50 and 100 mg, whereas the manual protocol was performed using 50 mg of starting material. DNA recovery was assessed by quantitative analysis of nuclear and mitochondrial DNA followed by mitochondrial sequencing using either the Precision ID mtDNA Whole-Genome Panel or a primer-extension-capture-based approach, depending on DNA quantity and degradation status.

By directly comparing quantitative DNA recovery and downstream sequencing performance, this study aimed to determine how both extraction strategies perform across samples of different preservation quality.

## 2. Materials and Methods

Tissue powder was prepared from skeletal material originating from 12 human individuals and 13 skeletal samples. All samples were milled into bone powder using a vibrating ball mill (MM2 ball mill, Retsch, Haan, Germany, or Laarmann Group BV, Roermond, The Netherlands) under controlled laboratory conditions at room temperature prior to DNA extraction. Throughout this manuscript, the term “individual” refers to a human donor, “skeletal sample” to a single bone or tooth specimen, “powder aliquot” to the portion of milled material used for a single DNA extraction, and “extract” to the DNA solution obtained from that extraction. Where applicable, replicate extracts were generated from separate powder aliquots of the same skeletal sample.

One individual (B3) was included in both parts of the study because sufficient remaining material was available to allow its analysis under the experimental conditions of Extraction Test 2. The inclusion of B3 in both experiments enabled an additional assessment of extraction performance using the same skeletal sample across two experimental setups, while recognizing that the experimental conditions differed between the two tests. The samples were already analysed in earlier studies, and the remaining powder, which had been stored frozen at −20 °C as backup material, was used in this study. Supplementary Table S1 provides an overview of the samples, including their historical age, the DNA extraction method used, and the handling time for each sample.

For the first part of the study, four teeth (T1–T4) and three bones (B1–B3) from different historical periods were used (dating from the early bronze age (c. 2200–1600 Before Common Era (BCE)) to the 7th and the 19th centuries (Supplementary Table S1). DNA from these samples was extracted with the Maxwell Forensic Sample Concentrator (FSC) (Promega, Madison, WI, USA) instrument and the Bone DNA Extraction Kit (Promega, Madison, WI, USA) using 50 mg (Max-50) and 100 mg (Max-100) of tissue powder each. Although the manufacturer recommends a starting input of 100 mg, we also used 50 mg as starting amount to allow direct comparison with the modified manual Dabney method (DAB [1]), which also uses 50 mg powder input.

Sample B1 was also tested using the automated DNA extraction method to compare the results to the earlier DAB extraction that produced a milk-coloured, oily lysate with a pellet that could not be fully separated despite repeated centrifugation.

For the second part of the study, five samples from an Austrian medieval cemetery [2] were selected. These included three teeth (T5–T7) and two bones (B4, B5) dating to the 5th/6th and 12th/13th centuries. Bone B3, which had already been included in Extraction Test 1, was also included in Extraction Test 2. The five samples were originally extracted between 2010 and 2013 using a modified phenol/chloroform/isoamyl alcohol (PCI) [3,4] and spin filter (SF) [5] extraction protocol. All samples were extracted in triplicate, with the exception of bone B3 and tooth T7, for which the available amount of remaining tissue powder was limited due to previous analyses. Therefore, these samples could not be processed under all extraction conditions in triplicate. Bone B3 was extracted using Max-50 and DAB, whereas tooth T7 was extracted using Max-100 and DAB. The remaining four samples were extracted in triplicate under all three extraction conditions (Max-100, Max-50, and DAB). The detailed scheme in Supplementary Table S2 depicts sample loading and extraction protocols.

DNA quantification was carried out using the SD quants method [10] for all samples. This method enables the simultaneous quantification of one nuclear and two mtDNA targets of varying target sizes (69 bp and 143 bp) in a single assay (Supplementary Table S3).

From the second study, one replicate from each triplicate set and each extraction method was selected for sequencing. The sequencing method depended on mtDNA quality and quantity. Samples were sequenced either using the amplicon-based Precision ID mtDNA Whole-Genome Panel (PID; Thermo Fisher Scientific, Waltham, MA, USA) or the primer extension capture method (PEC) [11]. The sequencing method was selected based on the mtDNA quantification results for both targets (Supplementary Table S4). For samples analysed by PEC, positions within the control region (CR) were targeted, whereas for samples analysed by PID, the entire mitochondrial genome was sequenced.

Sequencing was restricted to Extraction Test 2 samples to ensure direct comparability between extraction methods under identical experimental conditions. This includes the use of triplicates and consistent sample processing within the same experimental framework. Samples from Extraction Test 1 were not included in sequencing, as they were primarily used for initial method evaluation. In addition, comparison with previously generated Dabney data obtained in earlier studies was not fully appropriate due to the differences in experimental setup and sample processing.

In sample B4, one of the triplicates showed substantially higher quantification values than the other two. This discrepancy may indicate either contamination or a technical artefact during real-time PCR analysis. To further investigate this observation, the corresponding extract was included in the sequencing analysis. If present, such contamination could be revealed by sequencing the sample using an amplicon-based method. To investigate this further, two of the B4 triplicates were analysed using PEC: the replicate with high quantification values and one replicate with low quantification values. In addition, the replicate with high quantification values was sequenced using PID to compare haplotypes and assess potential contamination.

All sequencing data were analysed using the Torrent Suite Software (TSS) v 5.18.1 (Thermo Fisher Scientific, Waltham, MA, USA). Sequences were aligned to the Precision ID mtDNA rCRS, alignment was checked and mtDNA haplogroups were obtained by SAM2 provided through EMPOP V4, R14 ([12], https://empop.online/; [13]). Tertiary analysis was performed using the HIDGenotyper-2.3 (Thermo Fisher Scientific, Waltham, MA, USA) for samples sequenced with the PID mtDNA Whole-Genome panel. Per-position read depth (RD) values obtained from the sequencing data analysis were used to calculate the mean and median RD across all positions of the analysed region (control region and complete mitochondrial genome). All data analysis and graphical representation of the results was performed using Google Colab (Google LLC, Mountain View, CA, USA), a web-based computational platform for Python programming (https://colab.research.google.com/; accessed on 11 June 2025) and Microsoft Excel (Microsoft Corporation, Redmond, WA, USA).

## 3. Results and Discussion

In both parts of the study a total of 62 extracts from 13 skeletal samples derived from 12 human individuals, including one sample (B3) analysed in both Extraction Test 1 and Extraction Test 2, together with five extraction blanks, were quantified for nDNA and mtDNA. Quantification values are reported in Supplementary Table S3. Extraction blanks did not yield any detectable DNA. Four samples were processed in triplicate for all three extraction methods, and two samples in triplicate for two of the three methods (Supplementary Table S3b). The remaining samples were processed using a single approach (Supplementary Table S3a). The study comprised two parts.

For the first part of the study (Extraction Test 1), seven samples were used for which DAB quantification data had already been generated in earlier analyses, some of which have been published [14]. The previously generated DAB quantification data were included solely as reference values for comparison with the Maxwell extractions and were not reanalysed in the present study. Accordingly, Extraction Test 1 should be regarded as an exploratory comparative analysis intended to provide contextual information rather than a direct method comparison. A more robust comparison is presented in Extraction Test 2, where all extraction protocols were evaluated within the same experimental framework using newly generated extracts. For the current study, earlier prepared powder aliquots that served as backup material stored in the freezer were used. These samples, originally extracted between 2017 and 2023 using the DAB protocol, were subjected to extraction using the Maxwell (FSC) instrument with starting material amounts of 50 mg and 100 mg, respectively (Supplementary Table S2a). In this test, the quantitation results of the Maxwell extraction protocol were directly compared with the earlier data from DAB extraction protocols.

The second part of the study (Extraction Test 2) included a total of 48 DNA extracts from six samples. Again, remaining powder aliquots stored in the freezer from earlier projects were used for this test. Four samples were extracted in triplicates using all three protocols (DAB, Max-50 and Max-100), one sample was extracted using DAB and Max-100, and another sample using DAB and Max-50 (Supplementary Table S2b,c). All extracts were quantified and individual replicates sequenced to compare results between the protocols.

### 3.1. Quantification Results

#### 3.1.1. Quantification Results—Test 1

A direct comparison of all three protocols was feasible for samples B2, T1, T2, and T4. For samples B1 and T3, the previously obtained DAB results were generated from triplicate reactions (DAB-3 × 50 mg), in which three 50 mg lysates were pooled. Consequently, a direct comparison between the two samples extracted using the Maxwell system (Max-50 and Max-100) and the existing DAB quantification values was not appropriate because of differences in sample processing.

Instead, a comparative analysis was performed after normalization of the DAB values. For this purpose, the DAB quantification values were divided by three to account for the fact that three bone-powder replicates had been combined and processed through a single column, as described by Xavier et al. [1]. This approach is consistent with their findings, which indicate that combining three replicates yields DAB lysate quantification values in line with theoretical expectations. Thus, the adjusted DAB values allowed an approximate estimate for comparison with the Max-50 and Max-100 quantification results.

The final sample in this test, B3, was extracted in triplicate using DAB and Max-50 in Test 2. Therefore, the comparative analysis of this sample is presented exclusively in Section 3.1.2, “Quantification results: Test 2.”

**Figure 1:**
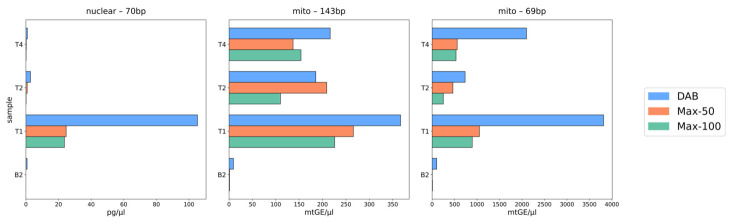
Quantification results from Test 1 for the nuclear target and the two mitochondrial targets (143 bp and 69 bp fragment).

A comparison of samples B2, T1, T2, and T4 is shown in Figure 1. DAB generally yielded the highest quantitative values for all four samples and across all three targets, with one exception: the 143 bp fragment in the mitochondrial quantification assay for sample T2. In this case, Max-50 produced the highest yield, with 209 mtGE/µL compared with 186 mtGE/µL for DAB (Supplementary Table S3).

**Figure 2:**
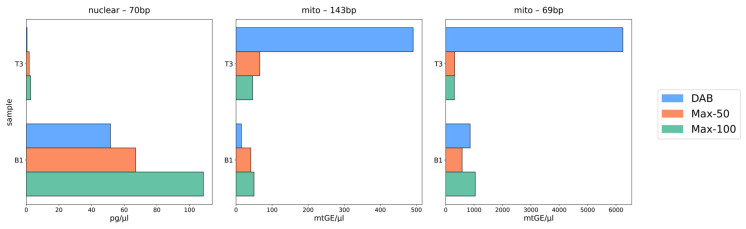
Quantification results from Test 1, comparing the Maxwell-extracted samples Max-50 and Max-100 with the previously extracted DAB sample (DAB-3 × 50 mg). Quantities for the DAB sample were normalized by dividing each measured value by 3.

Figure 2 presents the normalized quantities of the previously extracted DAB samples (DAB-3 × 50 mg), calculated by dividing each quantity value by three, in comparison with the values obtained from the two Maxwell-extracted samples, Max-50 and Max-100. In sample T3, DAB yielded markedly higher quantities of mitochondrial DNA than both Maxwell approaches. Among the Maxwell methods, Max-50 performed slightly better than Max-100 for both mitochondrial targets. However, all three methods produced only minimal amounts of nuclear DNA, with values close to background levels for this target.

In sample B1, Max-100 generally yielded higher quantitative values than the other methods across all three targets. Max-50 yielded higher values for nuclear DNA and for the larger mitochondrial target of 143 bp, whereas DAB produced higher quantities for the shorter mitochondrial target of 69 bp. Notably, the milky and oily appearance of the lysate from sample B1 did not appear to impair either the initial Dabney extraction or the subsequent Maxwell-based automated extraction. Both methods processed the sample successfully without any apparent issues.

#### 3.1.2. Quantification Results—Test 2

**Figure 3:**
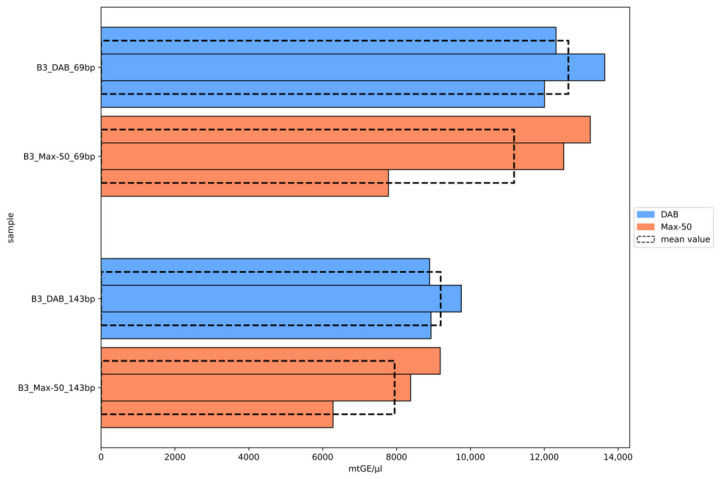
Quantification results from Test 2 for the two mitochondrial targets (143 bp and 69 bp fragment) in sample B3, comparing DAB and Max-50 extraction methods.

A considerable amount of mitochondrial DNA was measured in sample B3; therefore, this sample is presented in a separate figure (Figure 3). B3 was extracted using only DAB and Max-50. In the graph, the triplicates are plotted individually rather than as mean values; the mean for each method is additionally indicated by a dashed bar overlaid on the triplicate values. The results show very high quantities for both extraction methods across all triplicates. Although no clear advantage in overall DNA yield was observed between the two extraction methods for this sample, the Dabney method showed less variation among replicates than the Max-50 workflow.

**Figure 4:**
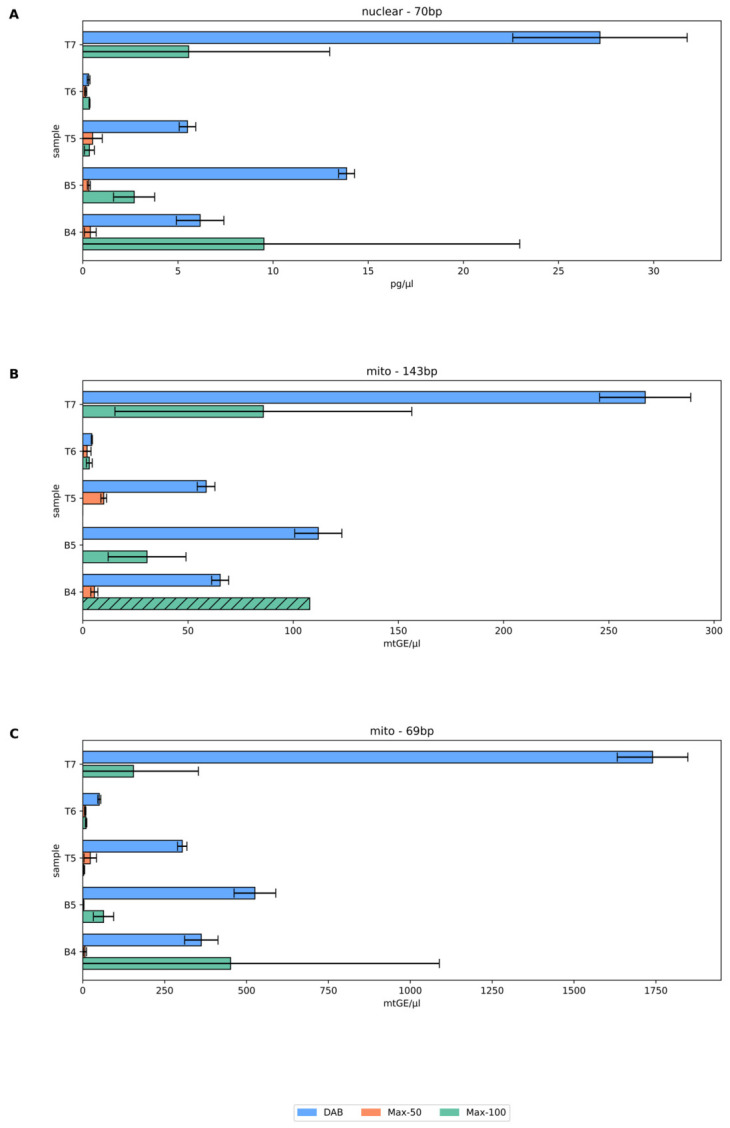
Quantification results from Test 2 for nuclear and mitochondrial DNA targets. (**A**) Quantification results for the nuclear DNA target (70 bp fragment) in samples B4, B5, T5, T6, and T7. (**B**) Quantification results for the mitochondrial DNA target with the 143 bp fragment. (**C**) Quantification results for the mitochondrial DNA target with the 69 bp fragment. Samples B4, B5, T5, and T6 were processed with all three extraction methods, while sample T7 was extracted using DAB and Max-100 only.

Figure 4 presents the quantification results for nuclear DNA (A, 70 bp) and mitochondrial DNA (B, 143 bp; C, 69 bp) in four samples processed in triplicate (B4, B5, T5, and T6), as well as sample T7, which was extracted using DAB and Max-100.

Across all targets, a clear trend was observed, with DAB generally producing higher quantification values than the two Maxwell-based extraction methods, Max-100 and Max-50, with the exception of sample B4, where Max-100 yielded higher quantities for all targets. However, for the 143 bp mtDNA target, only one of the triplicates produced a measurable value, whereas the other two were undetectable. Therefore, the corresponding bar in the graph is shown as dashed to indicate a potential outlier, as only one of the three values was measurable. In addition, the large standard deviation observed for the nuclear target and the shorter mtDNA target with Max-100 suggests that this isolated result may represent an outlier, potentially arising from a quantification artefact or a pipetting/handling issue during qPCR setup. To investigate this further, the corresponding extract was included in the sequencing analysis.

No clear trend was observed between Max-100 and Max-50. In some samples, such as T5, Max-50 produced higher values than Max-100, with Max-100 being almost undetectable for both mitochondrial targets and only minimally detectable for the nuclear target. In contrast, sample B5 showed higher values for Max-100, whereas Max-50 failed to produce measurable values for both mitochondrial targets. Sample T6 yielded very low values with all three methods, suggesting that it likely contained little to no detectable DNA. Sample T7 was extracted using only Max-100 and DAB. As observed for several of the other samples, DAB again yielded higher quantities than the Maxwell-based extraction method.

### 3.2. Sequencing Results

For sequencing, only samples from the second part of the study were used, in which triplicates of the different extraction methods had been processed. Sample selection was based on the detected mtDNA copy number, with thresholds determined according to the fragment-size requirements and sensitivity of each sequencing method.

For the PID protocol, which amplifies fragments of approximately 175 bp, a minimum of approximately 100–150 copies of the 143 bp target per reaction was considered necessary, although 200–300 copies were preferred for optimal sequencing results. In contrast, the PEC protocol is a hybridization-based assay and is therefore not constrained by amplicon size in the same way as PCR-based approaches. PEC relies on probe-based primer extension capture of target DNA. Successful enrichment depends on the physical availability of target fragments for hybridization. This method is typically used when low copy numbers of the 143 bp fragment indicate that PCR-based amplification is unlikely to succeed.

For PEC, the sample should not contain high amounts of the 143 bp target, particularly when large and small mtDNA fragments show similar quantification values. This pattern may indicate relatively intact DNA rather than highly degraded molecules. In samples with relatively intact DNA, the fragment length distribution may shift towards longer molecules, which can reduce the efficiency of hybridization-based enrichment due to reduced accessibility of target regions. If necessary, the DNA is sheared to obtain a more defined fragment-length distribution compatible with the assay. This step, however, may increase susceptibility to contamination with modern DNA, as contaminating DNA is also fragmented and may resemble endogenous degraded DNA. Due to these limitations and the considerable hands-on time, PEC is generally applied only to samples with low DNA quantities. Nevertheless, detection of at least some copies of the shorter 69 bp target remained a requirement to confirm the presence of endogenous DNA. Thus, the threshold decision was guided by method-specific requirements and DNA preservation patterns rather than by a fixed numerical value.

Supplementary Table S4 presents the quantification results for all targets, with samples selected for sequencing based on their mitochondrial DNA concentration highlighted in a specific colour.

Sample B4 showed an unusually high mtDNA copy number for the larger target in only one of its triplicates. As noted above, this outlier was suspected to result either from modern DNA contamination or from a quantification artefact. Therefore, extracts from this sample were sequenced using both the PEC and PID protocols.

Another sample, T7, was also analysed using both sequencing approaches. The larger target region showed relatively high quantification values, although not as high as those typically observed in samples that consistently yield successful results with these methods. Conversely, the shorter fragment showed a comparatively high value, raising the possibility that shearing, as described above, might be necessary for an effective capture assay. Overall, this sample represented a transitional case in which both sequencing approaches could potentially perform well, but in which challenges could also arise depending on the specific conditions.

In total, 20 samples were sequenced. Five samples, with values ranging from 108 to 9755 copies for the larger 143 bp mtDNA target, were sequenced using the PID protocol. Fifteen samples, with very low values for the 143 bp target, ranging from undetectable to 128 copies, but higher values for the shorter 69 bp target, ranging from 1 to 615 copies, were sequenced using the PEC protocol.

For sequencing-data analysis, the average read depth (RD) at each position was calculated and compared across all positions. In addition, the quality and purity of the sequencing data were carefully assessed for each sample. This evaluation focused on several key factors, including the identification of damage patterns, such as base substitutions or insertions/deletions (indels), which may indicate DNA degradation. These damage patterns were assessed by visual inspection to evaluate the extent of sample degradation and its potential impact on sequencing quality.

False-positive differences relative to the revised Cambridge Reference Sequence (rCRS) were also examined, as such discrepancies may indicate variant-calling artefacts or potential contamination. Examples of these damage patterns, including positions identified as false-positive differences relative to the rCRS as well as true differences from the rCRS, are shown in Supplementary Table S5.

Furthermore, strand bias was assessed to support the accuracy of the results, particularly in degraded samples. The level of background noise in the sequencing data was also evaluated, as excessive noise can obscure true signals and complicate interpretation. These metrics were integrated across all samples and extraction methods to provide a comprehensive evaluation of sequencing performance.

#### 3.2.1. Sequencing Results with the Precision ID Method

**Figure 5:**
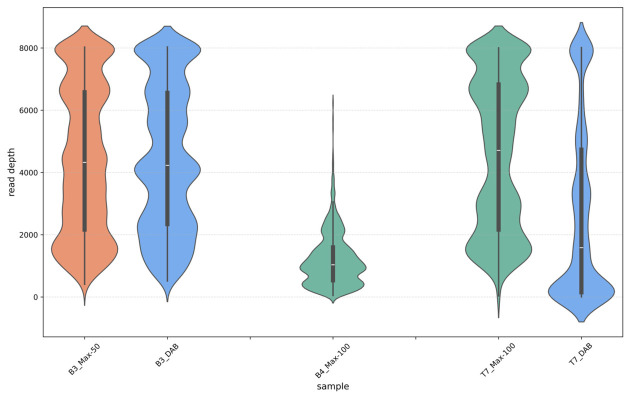
Comparison of read depth (RD) values across the five samples sequenced using the PID method. Violin plots illustrate the distribution of read depths across all positions of the mitochondrial sequences, showing the spread and density of RD values within each sample. Colors indicate the extraction method: orange, Max-50; blue, DAB; green, Max-100.

The PID sequencing results provided additional insights into the performance of the extraction methods. Figure 5 presents the read depth (RD) distribution across all positions for the five samples sequenced using the PID method, displayed as violin plots. This visualization shows the distribution of read depths across positions, highlighting the overall spread, density, and potential outliers within each sample, and thereby supporting the assessment of sequencing consistency and reliability. Supplementary Table S6 provides the median and mean read depth for the mitochondrial control region (CR) and the entire mitochondrial genome for all samples, together with the observed haplogroups and mitochondrial haplotypes. Unless stated otherwise, RD values reported throughout Section 3 refer to the mean read depth calculated across all positions of the respective mitochondrial sequence.

Sample B3 produced excellent results with both Maxwell and DAB, with RD values of 4384 and 4431, respectively, across the entire mitochondrial genome. Both methods successfully generated complete mitochondrial genome profiles with no observable damage patterns, highlighting the high quality of the sequencing data obtained from this sample.

An interesting result was observed for sample T7, for which the quantification data did not clearly predict the sequencing outcome. This sample was analysed using both sequencing approaches, PEC and PID. The decision to apply both strategies was based on the quantification data: although the amount of mitochondrial DNA detected for the 143 bp target was sufficient in principle, the overall quantity remained close to the lower range typically associated with successful PID sequencing. This raised uncertainty as to whether a complete mitochondrial genome profile could be generated successfully using the amplicon-based Precision ID mtDNA Whole-Genome Panel.

Overall, both extraction methods generated similar sequencing results for T7, with DAB showing slightly higher quantification values but no major differences in sequencing success. Following PID sequencing, both extracts yielded complete mitochondrial genome profiles, with slight but clearly distinguishable damage patterns observed. The Max-100 extract showed more balanced sequencing and a higher RD of 4614 compared with the DAB extract, which reached an RD of 2669. In the DAB extract, one of the amplification pools, PID Pool 2, underperformed.

One possible explanation for this discrepancy is a technical issue during library preparation for emulsion PCR on the Ion Chef system. In this workflow, the two library pools, Pool 1 and Pool 2, are quantified separately before being combined for subsequent emulsion PCR. A pipetting or measurement error at this step may have resulted in an insufficient amount of Pool 2 being added, which would explain its underperformance during sequencing.

PEC analysis of sample B4 yielded nearly identical results for both Max-100 extracts. These results are discussed in more detail in a later section. The PID results further supported this finding, as they showed no deviation in haplotype or overall data quality. PID sequencing yielded an average RD of 1194 and generated a complete mitochondrial genome profile with moderately high read depth, sufficient for comprehensive analysis. The sample exhibited only minor damage patterns, which appeared consistently as background noise. These findings suggest that the outlier observed during quantification was unlikely to have been caused by contamination and was more likely attributable to a technical artefact during qPCR quantification rather than a systematic issue.

#### 3.2.2. Sequencing Results with Primer Extension Capture Method

**Figure 6:**
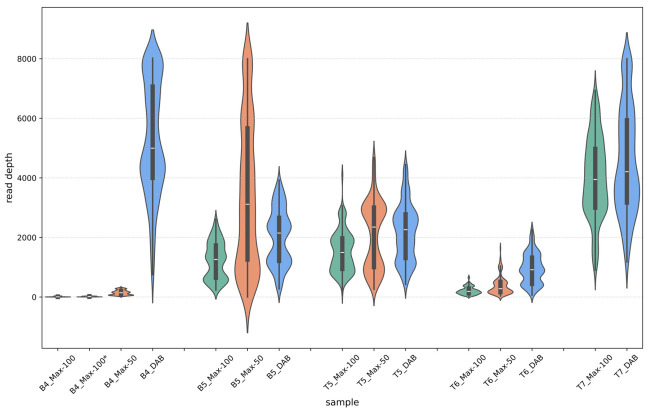
Comparison of read depth (RD) values across the 15 samples sequenced using the PEC method. Samples are ordered alphabetically by sample name. Colors indicate the extraction method: orange, Max-50; blue, DAB; green, Max-100. The Maxwell-extracted sample Max-100* is included for reference; its isolated value is likely an outlier, possibly due to contamination.

Figure 6 presents the read depth (RD) at each position for the 15 samples sequenced using the PEC method, with samples ordered alphabetically by sample name. Corresponding RD values, including both median and mean read depth for the CR as well as for the entire mitochondrial genome, as well as the observed haplogroups and mitochondrial haplotypes for each sample, are provided in Supplementary Table S6.

In the following section, each sample is discussed individually, with samples arranged sequentially from those exhibiting the most consistent sequencing performance to those showing progressively reduced performance. To facilitate detailed inspection, each sample is shown separately in an individual Supplementary Figure (Supplementary Figures S1–S5), displaying the corresponding read depth profiles for the different extraction approaches of this sample.

In sample T7, DAB exhibited a slightly higher RD (4597) than Max-100 (4019). However, this difference was minor with respect to RD and sequencing outcomes (Supplementary Figure S1). The sequencing results were comparable for both extraction methods, indicating similar sequencing outcomes under these conditions. Both samples showed damage patterns distributed across the entire mitochondrial genome. These patterns were clearly identifiable, with damage rates around 1–4% of the read depth, allowing for reliable differentiation from the rCRS and accurate variant calling in both cases.

Sample B5 revealed a noteworthy pattern in the comparison of extraction methods. Max-50 showed a higher RD of 3465, compared to DAB with 2014 and Max-100 with 1229 (Supplementary Figure S2). However, this increased RD was not accompanied by higher sequencing quality. The Max-50 extract exhibited a higher number of incorrectly called positions, including false positives that were clearly distinguishable due to a strong strand bias (Supplementary Table S5E). The DAB extract showed the highest sequencing quality in this sample, whereas the Max-100 extract also generated high-quality sequencing data. Both DAB and Max-100 displayed mild, evenly distributed damage patterns across the genome, which were clearly recognizable and did not impair the accuracy of the analysis.

For the extraction samples of T5, RD analysis revealed that DAB exhibited higher RD values (2172), closely followed by Max-50 (2097), whereas Max-100 showed lower values (1529), accompanied by notable outliers (Supplementary Figure S3). This trend was reflected in the sequencing results. The DAB extract yielded the highest read depth together with the fewest apparent sequencing artefacts for this sample, showing clearly identifiable differences relative to the rCRS, followed by Max-50. Max-100 showed lower data quality in this sample, coinciding with pronounced damage patterns. These damage patterns included regions with unclear base calls, where the T or A proportion ranged between 30% and 50%. Additionally, false-positive positions with strong strand bias were observed (Supplementary Table S5A). In contrast, Max-50 showed less apparent damage, with clearer positions and improved data interpretability. DAB, while displaying some damage patterns (approximately 1–4% of the read depth across the entire sample), showed lower levels of degradation than observed for both Maxwell methods, and this consistent damage did not appear to substantially interfere with the analysis.

In B4, the DAB extract yielded the highest read depth and the most favourable sequencing results for this sample, with an RD of 5293, compared to the other extraction methods. Max-50 followed with RD of 147, while Max-100 yielded the lowest RD values, ranging from 14 to 16 (Supplementary Figure S4). If the elevated DNA quantity had resulted from contamination, the sequencing results from Max-100 of the two PEC samples would be expected to differ, particularly with respect to RD and sequence quality. However, PEC analysis yielded identical results for both Max-100 extracts, with consistent RD values of 14 and 16, respectively, and no observable differences in sequencing quality.

For samples T6, DAB yielded the highest RD (959) with no outlier positions, compared to Max-100 with RD of 217 and Max-50 with 386 (Supplementary Figure S5). However, despite these advantages, the sequencing results remained poor, with all samples yielding uncertain outcomes due to the limited DNA quantity. Both Max-50 and DAB yielded higher read depths than Max-100, but overall, the results remained suboptimal, highlighting the challenges of working with degraded samples. All three samples showed strong signs of damage patterns. In particular, Max-50 and DAB exhibited some very pronounced regions that were difficult to analyse but could still be correctly called (Supplementary Table S5B–D). For Max-100, however, the differences from the rCRS were less clear, often appearing as mixed positions rather than distinct variants, as seen in the other two methods.

Overall, within the analysed sample set, the DAB extracts frequently yielded higher read depths and generally higher sequencing quality. The Max-50 workflow produced comparable results for several samples, whereas the performance of Max-100 varied depending on DNA preservation and sample characteristics. For samples with higher DNA quantities, Max-100 also generated reliable sequencing results.

## 4. Conclusions

The Maxwell instrument offers user-friendly handling with minimal hands-on time, whereas the Dabney method is more time-consuming and labor-intensive, and requires experience and practice. For relatively well-preserved samples, such as sample B3, which is approximately 200 years old, with well-preserved DNA, the results obtained with Maxwell and Dabney were fully comparable. However, for highly degraded samples, such as B4 and B5, which represent samples with limited DNA preservation, dating to the 5th/6th and 12th/13th centuries, respectively, the Dabney method generally yielded higher amounts of both nuclear and mitochondrial DNA.

It should be noted that part of the quantitative comparison was based on previously generated Dabney data, whereas the Maxwell extractions were performed in the present study using stored powder aliquots. Therefore, these historical data should be interpreted as contextual reference values rather than as a direct benchmark for extraction performance. Furthermore, the normalized values derived from pooled Dabney extracts provide only an approximate comparison with individually processed Maxwell extracts, as pooled and individually processed extracts do not represent fully equivalent experimental units. This should be considered when interpreting quantitative differences between the extraction methods. Accordingly, the strongest conclusions of this study are based on Extraction Test 2, where all extraction methods were evaluated under the same experimental framework using newly generated extracts.

Within the investigated sample set, the quantification results showed a clear trend towards higher DNA yields with the Dabney method, compared with the Maxwell-based extractions. The sequencing results generally confirmed this trend, although the differences between the two methods were less pronounced than in the quantification data.

Although the manufacturer recommends a starting input of 100 mg of tissue powder, reducing the input to 50 mg was associated with improved performance of the Maxwell workflow for severely degraded samples. This suggests that the observed differences between the extraction methods are driven by multiple factors, including starting material input, extraction chemistry, and sample preservation state. In addition, tissue type may represent another variable influencing DNA preservation and recovery efficiency. Although both teeth and bone samples were included in this study, a systematic evaluation of tissue-specific effects was beyond the scope of the present work and would require a larger sample set with controlled comparisons.

Despite these differences, the Maxwell workflow produced DNA extracts suitable for downstream analyses across the investigated samples. For several of the more challenging, severely degraded samples, reducing the starting material to 50 mg generally improved DNA recovery compared with the 100 mg workflow and, in some cases, yielded results approaching those obtained with Dabney. For less degraded samples, the standard 100 mg workflow generated sequencing results comparable to those obtained with the Dabney protocol when amplicon-based approaches such as the Precision ID mtDNA Whole-Genome Panel were applicable.

Overall, the results indicate that both extraction workflows can generate suitable DNA extracts for downstream analyses, although their relative performance may vary depending on the sample preservation state, extraction conditions, and the analytical approach applied. Further controlled studies using larger sample sets would be required to systematically evaluate the influence of these factors.

Due to the limited and heterogeneous nature of the dataset, no formal statistical tests were applied, as the assumptions required for robust inferential statistical analysis were not met.

## Data Availability

The original contributions presented in this study are included in the article/Supplementary Material. Further inquiries can be directed to the corresponding author.

## Supplementary Materials

The following supporting information can be downloaded at: https://www.mdpi.com/article/10.3390/genes17070842/s1. Figure S1. Read depth distribution for sample T7 across different extraction methods. Figure S2. Read depth distribution for sample B5 across different extraction methods. Figure S3. Read depth distribution for sample T5 across different extraction methods. Figure S4. Read depth distribution for sample B4 across different extraction methods. Figure S5. Read depth distribution for sample T6 across different extraction methods. Table S1. Description of samples, tests and quantification results. Table S2. Sample selection for DNA extraction procedures. Table S3. Quantification results. Table S4. Sample selection according to mitochondrial DNA quantification results. Table S5. Raw sequencing data screenshots. Table S6. Sequence summary metrics and haplotypes.
